# Development and validation a nomogram for predicting new-onset postoperative atrial fibrillation following pulmonary resection

**DOI:** 10.1186/s12893-024-02331-4

**Published:** 2024-01-31

**Authors:** Chuankai Zhang, Songsong Jiang, Jun Wang, Xianning Wu, Li Ke

**Affiliations:** 1 Department of Thoracic Surgery, The First Affiliated Hospital of USTC, Hefei, China; 2 Department of Thoracic Surgery, Division of Life Sciences and Medicine, The First Affiliated Hospital of USTC, University of Science and Technology of China, Anhui, Hefei, 230001 China; 3 Department of Cardiology, The Affiliated Anhui Provincial Hospital of Anhui Medical University, Hefei, China

**Keywords:** Pulmonary resection, New-onset postoperative atrial fibrillation (NOPAF), Prediction model, Nomogram

## Abstract

**Background:**

The new-onset postoperative atrial fibrillation (NOPAF) following pulmonary resection is a common clinical concern. The aim of this study was to construct a nomogram to intuitively predict the risk of NOPAF and offered protective treatments.

**Methods:**

Patients who underwent pulmonary resection between January 2018 and December 2020 were consecutively enrolled. Forward stepwise multivariable logistic regression analyses were used to screen independent predictors, and a derived nomogram model was built. The model performance was evaluated in terms of calibration, discrimination and clinical utility and validated with bootstrap resampling.

**Results:**

A total of 3583 patients who met the research criteria were recruited for this study. The incidence of NOPAF was 1.507% (54/3583). A nomogram, composed of five independent predictors, namely age, admission heart rate, extent of resection, laterality, percent maximum ventilation volume per minute (%MVV), was constructed. The concordance index (C-index) was 0.811. The nomogram showed substantial discriminative ability, with an area under the receiver operating characteristic curve of 0.811 (95% CI 0.758-0.864). Moreover, the model shows prominent calibration performance and higher net clinical benefits.

**Conclusion:**

We developed a novel nomogram that can predict the risk of NOPAF following pulmonary resection, which may assist clinicians predict the individual probability of NOPAF and perform available prophylaxis. By using bootstrap resampling for validation, the optimal discrimination and calibration were demonstrated, indicating that the nomogram may have clinical practicality.

## Introduction

The patients with sinus rhythm before pulmonary resection, but with new-onset atrial fibrillation after pulmonary resection are defined as NOPAF. NOPAF had no history of atrial fibrillation (AF), AF lasting > 30 seconds captured on a standard 12-lead electrocardiogram or cardiac monitor, or paroxysmal AF or atrial flutter intervened with pharmacological therapy or electrical resuscitation [1]. NOPAF remains a recognized surgical complication following thoracic surgery that has a significant impact on patient recovery and short-term or long-term outcomes [2]. The incidence of NOPAF in non-cardiac thoracic surgery was about 10%, which was most common on the second and third days after surgery [3]. Furthermore, NOPAF can seriously affect hemodynamics and results in treatment-related adverse events, ultimately leading to hypotension, heart failure, bleeding, drug toxicity, stroke [4, 5] and myocardial infarction [6]. In addition, NOPAF may place a heavy burden on patient care due to increased length of stay and resource consumption [7].

NOPAF is a sophisticated pathological reaction, and its specific mechanism has not yet been elucidated [8, 9]. However, a large amount of evidence currently suggested that NOPAF may be related to inflammation, myocardial ischemia, sympathetic activation, and so on [10, 11]. Although there have been numerous studies exploring the exact mechanisms of NOPAF, these mechanisms are still far from being elucidated. Therefore, identification of independent risk factors of NOPAF and targeted prevention are imperative to reduce the morbidity and mortality of NOPAF.

While there have been many reports regarding the predictive factors, prevention, and prognosis of NOPAF, there is still a lack of accurate predictive models for NOPAF after pulmonary resection. Some studies have found that the main risk factors of NOPAF include age increase, male, high body mass index (BMI), hypertension, diabetes, coronary artery disease and other basic diseases [12]. In addition, preoperative control of heart rate or rhythm seems to be an effective preventive measure for preventing NOPAF [13, 14]. Some studies have shown that changes in operation methods are also important predictors for NOPAF. The surgical scope, minimally invasive or thoracotomy surgery were considered as important influencing factors for NOPAF in lung surgery [15, 16]. Furthermore, the extent of lung resection has also been related to NOPAF. The incidence of NOPAF in patients received pneumonectomy is higher than patients received lobar and sub-pulmonary lobe resections [17]. However, there is still controversy about the risk factors for the development of NOPAF after pulmonary resection, and there is a lack of an intuitive, simple, and practical method for predicting the probability of NOPAF in each patient.

Therefore, identifying patients who are prone to NOPAF and implementing targeted prophylaxis may be cost-effective. Unfortunately, a simple, convenient and effective tool to predict NOPAF following lung resection has not yet been identified. The nomogram has been proved to be a reliable predictive tool with the ability to generate an individual probability of a clinical event by graphically representing the effect of each predictor on the outcome [18]. In this study, we employed a large dataset to investigate the clinical characteristics of patients with NOPAF and developed an efficient nomogram to predict the risk of NOPAF probability.

## Methods

This retrospective, observational study was approved by the Institutional Review Board of the First Affiliated Hospital of University of Science and Technology of China (USTC) (registration number 2023-RE-187) and individual informed consent for this retrospective analysis was waived. The study was conducted in accordance with the Declaration of Helsinki (as revised in 2013).

### Study population

This project was a cohort study of patients who underwent lung resection at the First Affiliated Hospital of USTC from January 2018 to December 2020. The inclusion criteria were as follows: patients with pulmonary malignancies or benign lesions underwent pulmonary resection; patients aged >18 years; preoperative electrocardiogram has been completed. The exclusion criteria were as follows: patients with incomplete perioperative data; preoperative diagnosis of AF or atrial flutter. Ultimately, a total of 3583 patients were enrolled in the retrospective study, which was approved by the Institutional Ethics Committee. All patients were used to develop the nomogram prediction model, while the bootstrap resampling was applied to validate the model.

### Data collection

Demographics including age, sex, BMI, cardiovascular disease (coronary heart disease, heart valve disease, etc.), hypertension, diabetes, cerebral infarction, smoking history (past or current smoker and never smoker) and drinking history, were retrospectively documented from electronic medical records. Preoperative laboratory data for all patients were the last recorded data before surgery. Data of preoperative evaluations including electrocardiogram (heart rate and rhythm), percent forced expiratory volume in 1 second (%FEV1), %MVV, left ventricular ejection fraction (LVEF), left atrial anteroposterior diameter (LAD), left ventricular end-diastolic volume (LVEDV), left ventricular end-systolic volume (LVESV), preoperative potassium ion, preoperative sodium ion, preoperative calcium ion, preoperative magnesium ion, percent preoperative neutrophils (%), percent preoperative lymphocytes (%), preoperative platelet count, preoperative hemoglobin, and New York Heart Association (NYHA) classification were also recorded. Perioperative surgical data were also collected including invasiveness (thoracotomy or video-assisted thoracic surgery (VATS)), surgical duration, resected segment (upper, middle, lower, others), laterality (right, left, right and left), extent of resection (pneumonectomy, lobectomy, lobectomy combined with segmentectomy or wedge resection(complex resection), minor resection), histological type (adenosquamous carcinoma, adenocarcinoma, squamous carcinoma, others), diameter of lesion, length of hospital stay, number of lymph node dissected, intraoperative blood loss.

### Developing the nomogram

The data of NOPAF and non-NOPAF were compared in our cohort, and the screening criterion for risk factors for NOPAF was *P* value < 0.1 in univariable analysis. All potential variables included in the multivariable analysis were subjected to a correlation matrix for analysis of multicollinearity. Subsequently, multivariate logistic stepwise regression (likelihood ratio) analysis was performed to further screen for significant risk factors for NOPAF using a *P*-value < 0.05 as the standard. Based on the multivariate analysis results, a nomogram was established using the rms package in R. Bootstrap resampling was performed to validate the accuracy of the nomogram model. The area under the receiver operating characteristic (ROC) curve (AUC) was used to assess the discriminative ability of the model. The calibration evaluated how close the predicted probabilities agree with the actual results [19]. Decision curve analysis (DCA) shows the standardized net benefit relative to the risk threshold probability and is used to assess the clinical utility of the model [20].

### Statistical analysis

Measurement data were expressed as mean ± standard deviation (SD) or median (25th, 75th percentiles) in case of normal or non-normal distribution. Student’s t-test or Mann-Whitney U-test were employed to compare the differences between two groups. Categorical variables were reported as counts (percentage) and compared with Pearson chi-square test or Fisher exact test. Univariate logistic regression analysis was used to identify potential predictors of NOPAF. All variables with *p* < 0.1 in univariate analysis were included in the multivariable logistic analysis. Variables with *P* < 0.05 in multivariable analysis were selected into the final nomogram model. Statistical analyses were performed with SPSS software, version 26.0 (SPSS Inc., Chicago, IL, USA) and R language (version 4.1.3). A two-tailed *P* < 0.05 indicated statistical significance.

## Results

### Demographics and clinical characteristics

From January 2018 to December 2020, A total of 6412 patients received thoracic surgery in the First Affiliated Hospital of University of Science and Technology. Of these patients, 1566 patients were excluded for undergoing non-pulmonary resection surgery, 163 patients under the age of 18 years were also excluded, 13 patients with a history of preoperative atrial fibrillation were excluded, and 1081 patients were also excluded due to incomplete perioperative data. Finally, 3583 eligible patients were enrolled, of whom 54 (1.507%) suffered from NOPAF.

The majority of patients (3401) underwent VATS, and 182 patients received thoracotomy. These patients were divided into two groups (patients without NOPAF and patients with NOPAF) according to the presence or absence of NOPAF. Clinical characteristics of both with NOPAF and without NOPAF were demonstrated in Table 1. Patients with NOPAF were significantly older than patients without NOPAF (*p* < 0.001). The age of NOPAF group was 66.0 ± 8.6 years, and 1709 (47.697%) were female patients, 61(1.702%) patients with cerebral infarction. Of these patients, 2128 underwent right pulmonary resection, 1418 underwent left pulmonary resection, and 37 received bilateral pulmonary resection. Patients in the NOPAF group had a significantly longer hospital stay compared to the non-NOPAF group (*p* < 0.001). Compared with the NOPAF group, the admission heart rate in the non-NOPAF group significantly increased (*p* = 0.008). The number of lymph nodes dissected was significantly higher in the NOPAF group (16.1 ± 10.2) compared to the non-NOPAF group (9.1 ± 9.9) (*p* < 0.001). The %MVV was significantly higher in the non-NOPAF group than in the NOPAF group (*p* = 0.026).

**Table 1:** Patient characteristics compared by presence and absence of NOPAF

**Characteristic**	**Without NOPAF (*N*=3529)**	**With NOPAF (*N*=54)**	***P***
Sex			0.011
Female	1693 (48%)	16 (29.6%)	
Male	1836 (52%)	38 (70.4%)	
Age (years), Mean ± SD	57.4 ± 12.3	66.0 ± 8.6	< .001
BMI (kg/m^2^), Mean ± SD	23.4 ± 3.3	22.6 ± 3.7	0.068
NYHA classification			0.112
1	857 (24.3%)	7 (13%)	
2	2561 (72.6%)	44 (81.5%)	
3	111 (3.1%)	3 (5.6%)	
Admission heart rate (beats/min), Mean ± SD	76.1 ± 10.5	72.2 ± 10.9	0.008
Current smoking			0.338
No	3158 (89.5%)	51 (94.4%)	
Yes	371 (10.5%)	3 (5.6%)	
Drinking			0.908
No	3353 (95%)	52 (96.3%)	
Yes	176 (5%)	2 (3.7%)	
Hypertension			0.752
No	2915 (82.6%)	46 (85.2%)	
Yes	614 (17.4%)	8 (14.8%)	
Diabetes			1
No	3309 (93.8%)	51 (94.4%)	
Yes	220 (6.2%)	3 (5.6%)	
Heart disease			1
No	3490 (98.9%)	53 (98.1%)	
Yes	39 (1.1%)	1 (1.9%)	
Cerebral infarction			0.094
No	3471 (98.4%)	51 (94.4%)	
Yes	58 (1.6%)	3 (5.6%)	
FEV1 (%), Mean ± SD	78.2 ± 13.9	71.7 ± 19.7	0.018
MVV (%), Mean ± SD	99.5 ± 20.2	93.4 ± 19.2	0.026
LVEF, Mean ± SD	68.3 ± 12.9	65.7 ± 9.4	0.051
Left Atrial Diameter (mm)			0.291
≥ 40	506 (14.3%)	11 (20.4%)	
< 40	3023 (85.7%)	43 (79.6%)	
LVEDV (ml), Mean ± SD	117.2 ± 24.8	123.4 ± 24.8	0.066
LVESV (ml), Mean ± SD	37.7 ± 11.8	38.1 ± 18.4	0.888
Kalium (mmol/L), Mean ± SD	4.3 ± 6.8	4.2 ± 0.4	0.529
Sodium (mmol/L), Mean ± SD	145.6 ± 234.6	141.0 ± 3.1	0.25
Calcium (mmol/L), Mean ± SD	2.3 ± 0.1	2.3 ± 0.2	0.836
Magnesium (mmol/L), Mean ± SD	1.0 ± 3.5	0.9 ± 0.1	0.042
% neutrophils, Mean ± SD	65.4 ± 95.9	63.6 ± 13.4	0.455
% lymphocytes, Mean ± SD	26.5 ± 11.8	25.6 ± 13.0	0.586
Hemoglobin (g/L), Mean ± SD	134.9 ± 27.2	140.3 ± 33.8	0.244
Platelet count (10^9^/L), Mean ± SD	215.0 ± 87.9	217.9 ± 99.9	0.814
Surgical duration (min), Mean ± SD	133.8 ± 75.4	162.2 ± 62.1	0.006
Invasiveness			< .001
VATS	3358 (95.2%)	43 (79.6%)	
Thoracotomy	171 (4.8%)	11 (20.4%)	
Resected segment			0.244
Middle	308 (8.7%)	2 (3.7%)	
Upper	1960 (55.5%)	37 (68.5%)	
Others	64 (1.8%)	1 (1.9%)	
Lower	1197 (33.9%)	14 (25.9%)	
Laterality			< .001
Right	2104 (59.6%)	24 (44.4%)	
Left	1393 (39.5%)	25 (46.3%)	
Right and left	32 (0.9%)	5 (9.3%)	
Extent of resection			< .001
Minor resection	1584 (44.9%)	5 (9.3%)	
Complex resection	54 (1.5%)	1 (1.9%)	
Lobectomy	1858 (52.6%)	42 (77.8%)	
Pneumonectomy	33 (0.9%)	6 (11.1%)	
Histological type			< .001
Others	884 (25%)	13 (24.1%)	
Squamous carcinoma	344 (9.7%)	15 (27.8%)	
Adenocarcinoma	2296 (65.1%)	26 (48.1%)	
Adenosquamous	5 (0.1%)	0 (0%)	
Diameter of lesion (cm), Mean ± SD	2.3 ± 2.8	3.3 ± 2.1	0.001
Number of lymph nodes dissected, Mean ± SD	9.1 ± 9.9	16.1 ± 10.2	< .001
Length of hospital stay (days), Mean ± SD	9.6 ± 5.0	15.4 ± 8.7	< .001
Blood loss (ml), Mean ± SD	54.2 ± 118.3	73.3 ± 110.7	0.237

*BMI* body mass index, *NYHA* New York Heart Association, *%FEV1* percent forced expiratory volume in 1 second, *%MVV* percent maximum ventilation volume per minute, *LVEF* left ventricular ejection fraction, *LVEDV* left ventricular end-diastolic volume, *LVESV* left ventricular end-systolic volume, *VATS* video-assisted thoracic surgery, *NOPAF* new-onset postoperative atrial fibrillation

### Predictors of NOPAF

The univariate analysis identified the following variables associated with NOPAF (*P*<0.1): sex, age, BMI, NYHA classification, admission heart rate, surgical duration, invasiveness, extent of resection, laterality, diameter of lesion, number of lymph node dissected, history of cerebral infarction, %FEV1, %MVV, %LVEF, LVEDV (Table 2). Furthermore, using multivariable logistic regression analysis, the results showed that 5 variables: age (OR, 1.04; 95% CI, 1.00-1.08; *P* < 0.05), admission heart rate (OR, 0.97; 95% CI, 0.94-1.00; *P* < 0.05), extent of resection-lobectomy (OR, 5.41; 95% CI, 1.90-15.43; *P* < 0.05), extent of resection-pneumonectomy (OR, 22.97; 95% CI, 4.73-111.62; *P* < 0.001), laterality (right and left) (OR, 30.07; 95% CI, 8.02-112.75; *P* < 0.001), and %MVV (OR, 0.98; 95% CI, 0.96-1.00; *P* < 0.05) were independent predictors of NOPAF after pulmonary resection (*P*< 0.05; Table 3).

**Table 2:** Univariate logistic regression of NOPAF presence

**Variable**	**OR(95% CI)**	***P***
Sex, male vs. female	2.19 (1.22-3.94)	0.009
Age, years	1.07 (1.05 -1.11)	<.001
BMI, kg/m^2^	0.92 (0.84 -1.00)	0.063
NYHA classification		
2 vs. 1	2.10 (0.94 - 4.69)	0.069
3 vs. 1	3.31 (0.84 -12.98)	0.086
Admission heart rate, beats/min	0.96 (0.94 - 0.99)	0.008
Surgical duration, min	1.00 (1.00 -1.01)	0.009
Resected segment		
Upper vs. middle	2.91 (0.70 -12.12)	0.143
Others vs. middle	2.41 (0.22 - 26.94)	0.476
Lower vs. middle	1.80 (0.41-7.97)	0.438
Invasiveness, thoracotomy vs. VATS	5.02 (2.55 - 9.91)	< .001
Extent of resection		
Complex resection vs. minor resection	5.87 (0.67 - 51.08)	0.109
Lobectomy vs. minor resection	7.16 (2.83 -18.14)	< .001
Pneumonectomy vs. minor resection	57.60 (16.74 - 198.22)	< .001
Laterality		
Left vs. right	1.57 (0.90 - 2.77)	0.115
Right and left vs. right	13.70 (4.92 - 38.17)	< .001
Diameter of lesion, cm	1.03 (1.00 -1.06)	0.061
Number of lymph nodes dissected	1.06 (1.03 - 1.08)	< .001
Blood loss, ml	1.00 (1.00 -1.00)	0.249
Current smoking, yes vs. no	0.50 (0.16 -1.61)	0.246
Drinking, yes vs. no	0.73 (0.18 - 3.03)	0.668
Hypertension, yes vs. no	0.83 (0.39 - 1.76)	0.619
Diabetes	0.89 (0.27 - 2.86)	0.838
Heart disease	1.69 (0.23 - 12.52)	0.608
Cerebral infarction	3.52 (1.07 - 11.61)	0.03
% FEV1	0.97 (0.96 - 0.99)	< .001
% MVV	0.98 (0.97 - 1.00)	0.026
LVEF	0.95 (0.92 - 0.98)	0.002
Left Atrial Diameter, more than 40 vs. less than 40, mm	0.65 (0.34 -1.28)	0.214
LVEDV, ml	1.01 (1.00 -1.02)	0.066
LVESV, ml	1.00 (0.98 - 1.03)	0.828
Kalium, mmol/L	1.00 (0.93-1.07)	0.935
Sodium, mmol/L	1.00 (0.96 - 1.03)	0.887
Calcium, mmol/L	1.27 (0.20 - 8.12)	0.804
Magnesium, mmol/L	0.51 (0.03 - 9.72)	0.650
% Neutrophils	1.00 (0.98 - 1.02)	0.902
% Lymphocytes	0.99 (0.97 - 1.02)	0.586
Hemoglobin, g/L	1.00 (1.00 - 1.01)	0.215
Platelet count, 10^9^/L	1.00 (1.00 - 1.00)	0.814

*BMI* body mass index, *NYHA* New York Heart Association, *%FEV1* percent forced expiratory volume in 1 second, *%MVV* percent maximum ventilation volume per minute, *LVEF* left ventricular ejection fraction, *LVEDV* left ventricular end-diastolic volume, *LVESV* left ventricular end-systolic volume, *VATS* video-assisted thoracic surgery, *NOPAF* new-onset postoperative atrial fibrillation

**Table 3:** Multivariable logistic regression of NOPAF presence

**Variable**	**OR(95% CI)**	***P***
Sex, male vs. female	1.71 (0.77 - 3.81)	0.188
Age, years	1.04 (1.00 -1.08)	0.046
BMI, kg/m^2^	0.93 (0.85 -1.03 )	0.166
NYHA classification		
2 vs.1	1.48 (0.63 - 3.45)	0.369
3 vs.1	1.74 (0.41 -7.34)	0.453
Admission heart rate, beats/min	0.97 (0.94 -1.00)	0.043
Surgical duration, min	1.00 (1.00 -1.00)	0.542
Invasiveness, thoracotomy vs. VATS	1.87 (0.73 - 4.76)	0.190
Extent of resection		
Complex resection vs. minor resection	4.68 (0.49 - 44.98)	0.182
Lobectomy vs. minor resection	5.41 (1.90 -15.43 )	0.002
Pneumonectomy vs. minor resection	22.97 (4.73 -111.62)	< .001
Diameter of lesion, cm	0.99 (0.85 - 1.15)	0.894
Laterality		
Left vs. right	1.54 (0.83 - 2.83)	0.169
Right and left vs. right	30.07 (8.02 -112.75)	< .001
Number of lymph nodes dissected	1.02 (0.99 -1.04 )	0.242
Cerebral infarction, yes vs. no	2.88 (0.82 -10.17)	0.100
% FEV1	0.98 (0.97-1.00)	0.086
% MVV	0.98 (0.96 - 1.00)	0.028
LVEF	0.97 (0.93 -1.01)	0.114
LVEDV, ml	1.00 (0.99 - 1.02)	0.602

*BMI* body mass index, *NYHA* New York Heart Association, *%FEV1* percent forced expiratory volume in 1 second, *%MVV* percent maximum ventilation volume per minute, *LVEF* left ventricular ejection fraction, *LVEDV* left ventricular end-diastolic volume, *LVESV* left ventricular end-systolic volume, *VATS* video-assisted thoracic surgery, *NOPAF* new-onset postoperative atrial fibrillation

### Building the nomogram

**Fig. 1:**
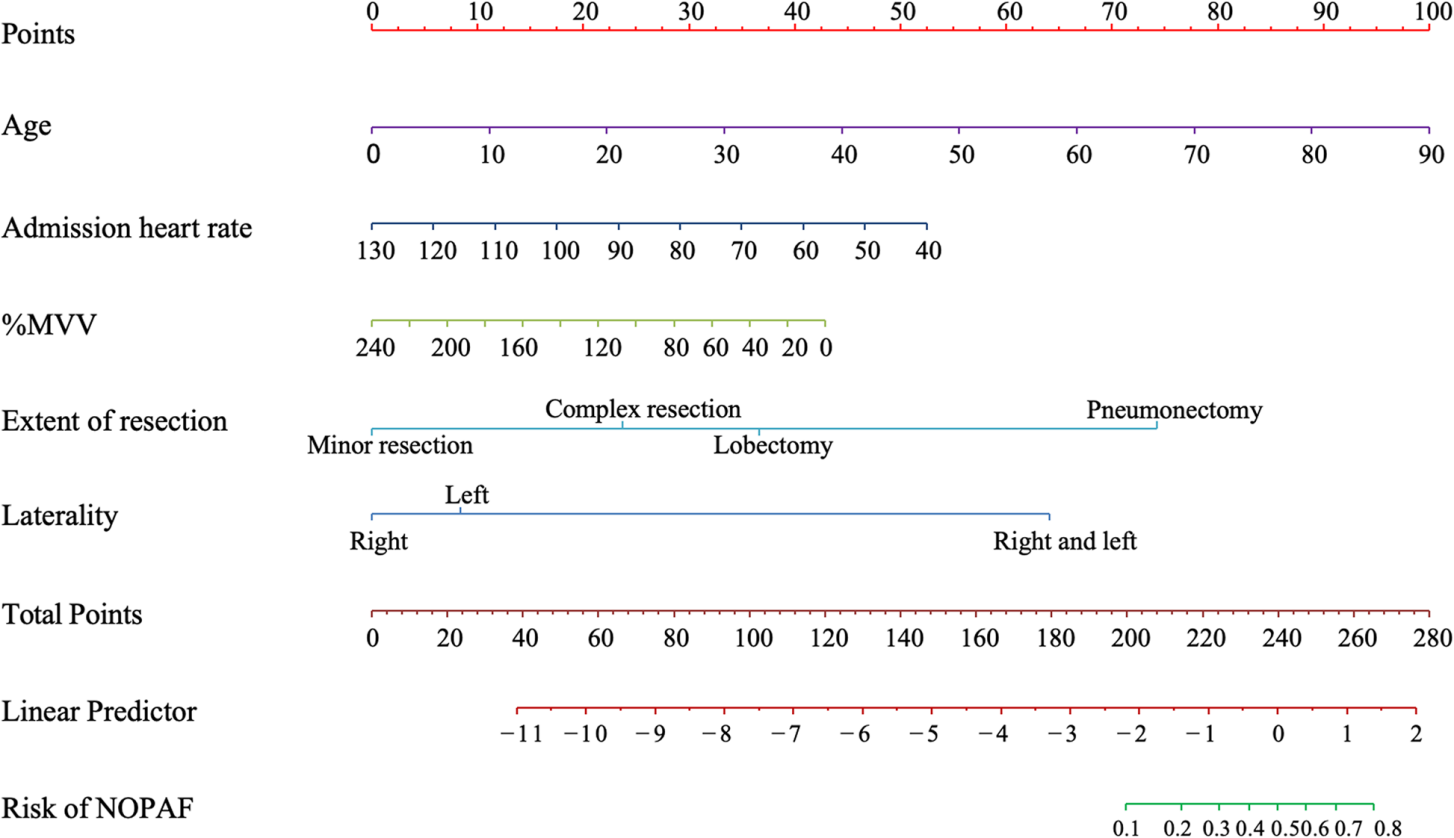
Nomogram predicting the risk of NOPAF after pulmonary resection. The different values of each variable correspond to different positions in the nomogram. Draw a line from the position of each variable to its corresponding point axis to obtain the point value of that variable. Calculate the number of points for different variables and sum them to obtain the total score. Based on the total score axis, the total score can be converted into the prediction probability of NOPAF. NOPAF, new-onset postoperative atrial fibrillation

A nomogram was established with the 5 independent predictors described above for predicting the probability of NOPAF after pulmonary resection (Fig. 1). In the nomogram, each variable was assigned to a point between 0 and 100, and the higher the total score, the higher the probability of NOPAF after pulmonary resection.

### Validation and calibration of the nomogram

**Fig. 2:**
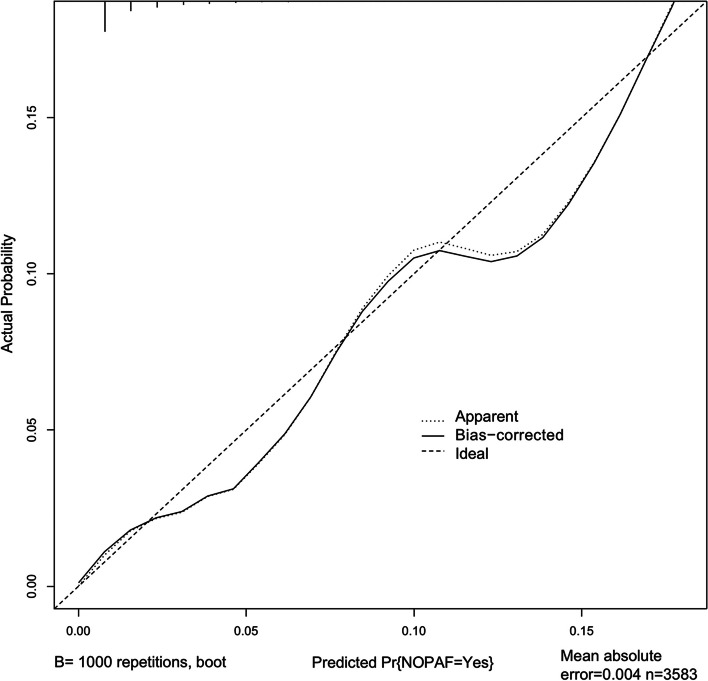
The calibration curves for the nomogram. The X-axis represents the predicted probability of NOPAF and the Y-axis represents the actual probability of NOPAF. Perfect prediction corresponds to the diagonal

**Fig. 3:**
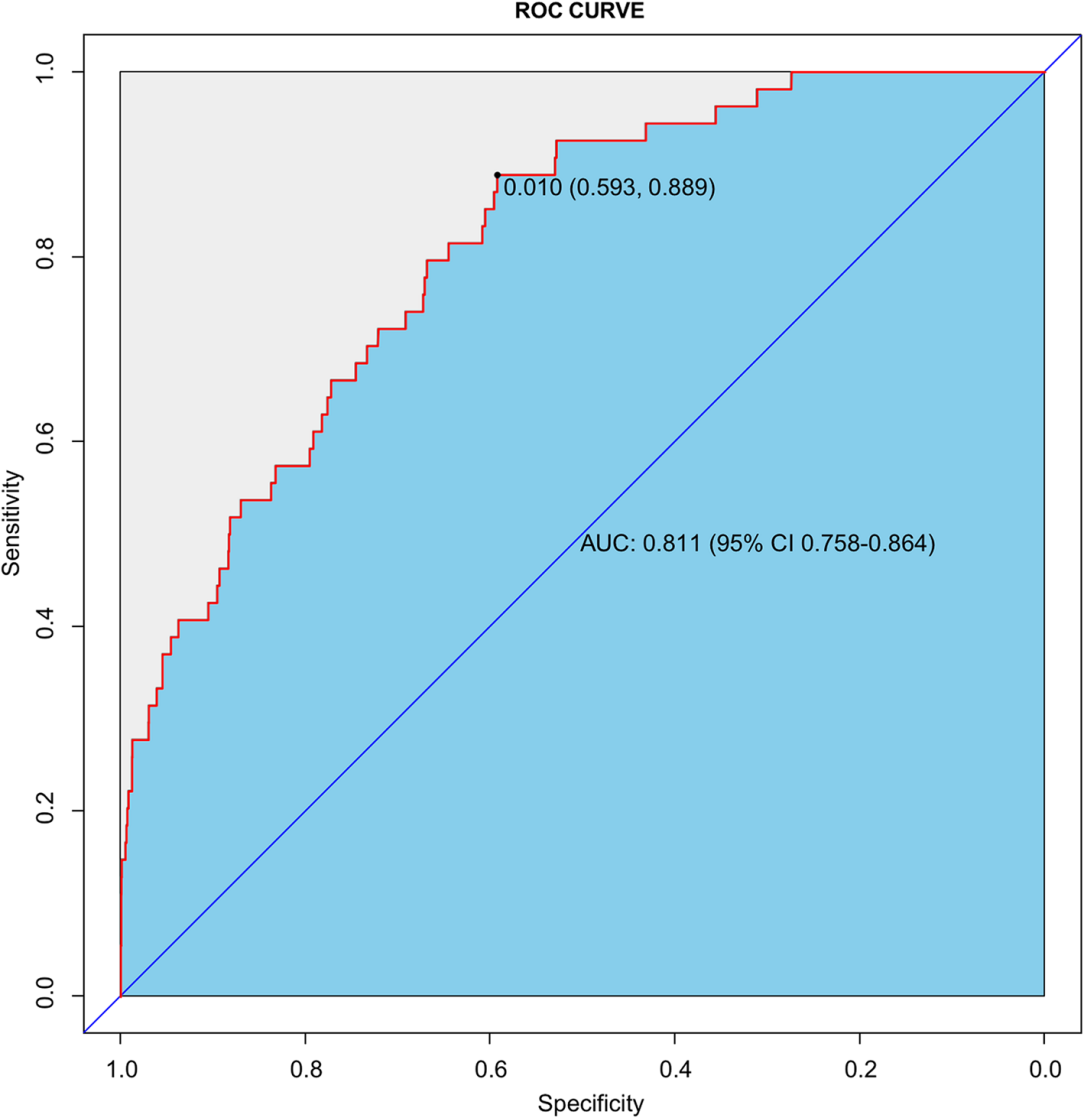
ROC for discrimination of the nomogram. The area under the ROC curve (AUC) was 0.811 (95% CI 0.758-0.864). ROC, receiver operating characteristic

**Fig. 4:**
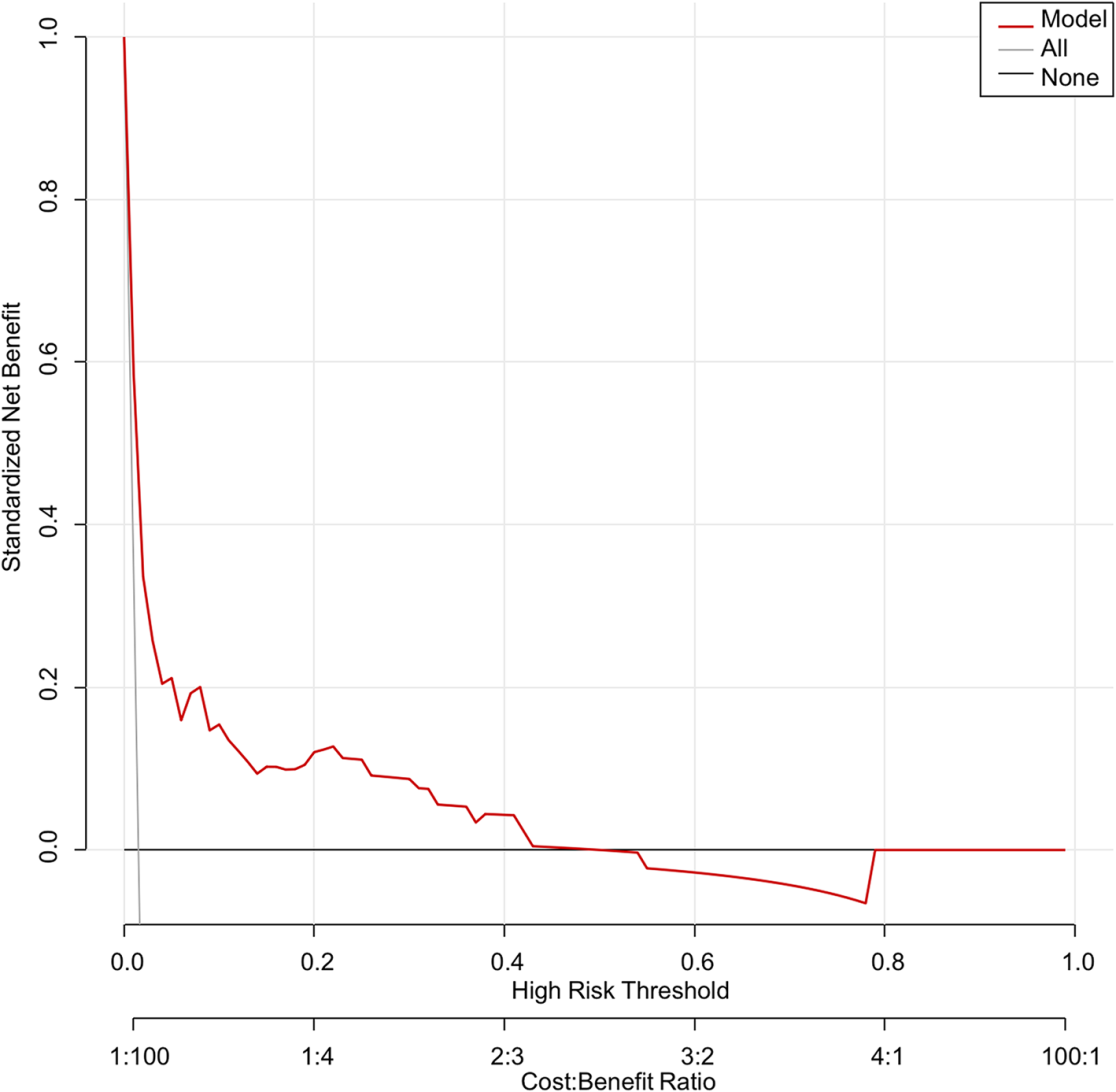
Decision curve for prediction of NOPAF for pulmonary resection. Decision Curve Analysis (DCA) is used to predict and estimate clinical usefulness and net benefits

**Fig. 5:**
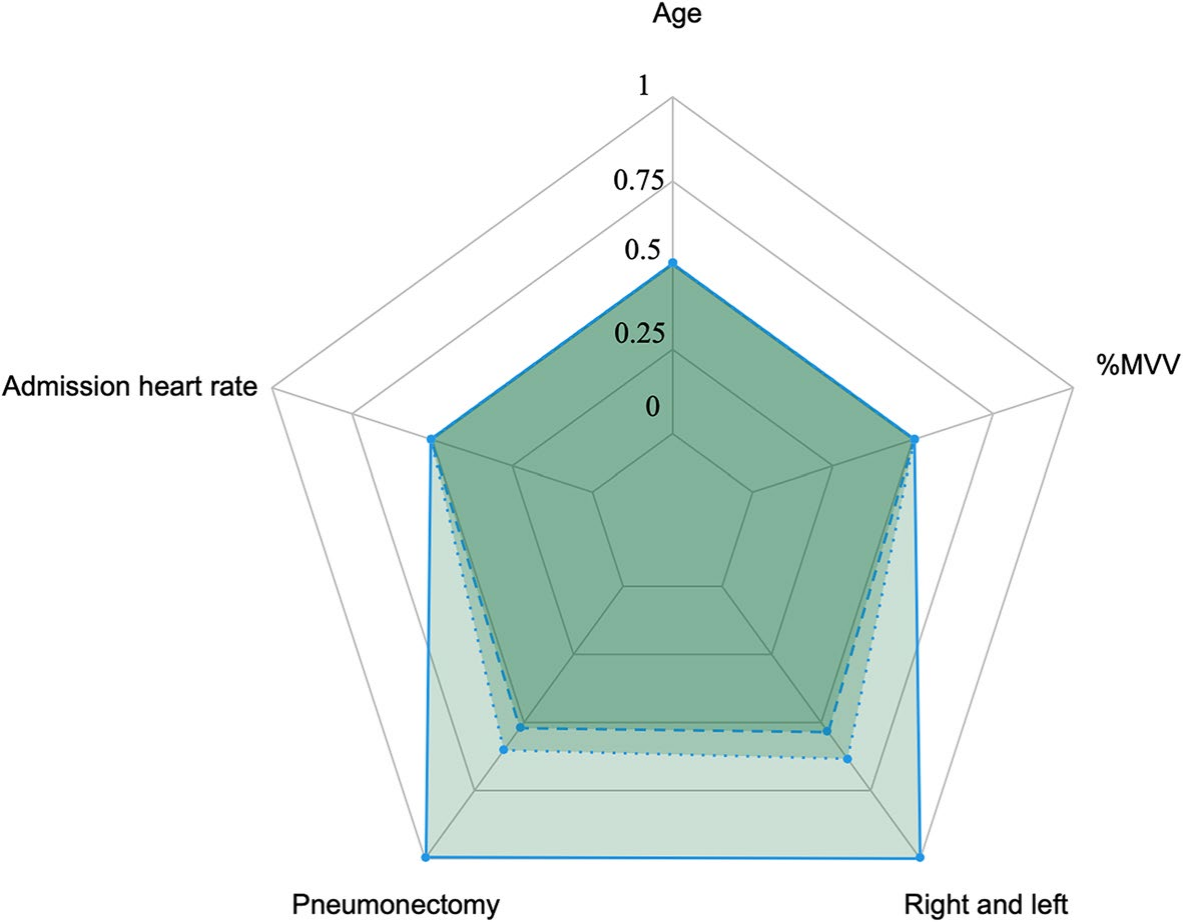
Radar charts were used to determine the importance of the 5 predictor variables

Calibration curve of nomogram was employed to provide the agreement between the predicted and observed results. The calibration curve of our model demonstrated a good agreement between the nomogram prediction and actual observation (Fig. 2). The discriminant degree of the nomogram was appraised by the ROC curve (AUC=0.811, 95% CI 0.758-0.864) (Fig. 3). DCA was applied in this study to evaluate the clinical utility of the nomogram (Fig. 4). This figure indicated that using this model to predict NOPAF and implementing appropriate interventions may be more beneficial than the initial treatment strategy. Construct a radar chart to determine the importance of the 5 predictive variables (Fig. 5).

## Discussion

An effective method for identifying high-risk patients with NOPAF can achieve targeted prevention strategies and avoid the risk of drug side effects and extra costs for the pulmonary resection population. In this situation, we developed a simple intuitive statistical prediction model that quantified the risk of NOPAF following pulmonary resection, which may help clinician making treatment recommendations. By incorporating five independent variables provided by multivariate logistic regression, namely age, admission heart rate, extent of resection, laterality, and %MVV, the nomogram showed well calibration, discrimination, and clinical utility.

AF often occurs following pulmonary resection and is generally referred to as postoperative atrial fibrillation (POAF) [21]. POAF may remarkably increase morbidity, mortality and hospital costs following thoracic surgery in the short and long term [22]. Our total incidence of NOPAF is 1.507% (54/3583), slightly lower than the incidence reported in previous studies, which estimated the incidence rate to be between 4% and 37% [22, 23]. The main reason for this situation may be due to, on the one hand, the high proportion of minimally invasive surgeries, on the one hand, and the large number of minor resections such as wedge resections and segmental resections in our cohort.

Our findings reaffirmed the importance of increasing age [24], extent of resection and pneumonectomy in the development of NOPAF after pulmonary resection. Similar to us, Ziad Mansour et al reported that the extent of pulmonary resection has also been related to NOPAF, and the incidence of NOPAF in patients with pneumonectomy has been higher than that with lobectomy or sublobectomy [25]. This conclusion is consistent with our results. Our analysis suggested that the incidence of NOPAF was significantly higher with pneumonectomy and lobectomy compared with minor resection. Recent researches with relatively large sample have demonstrated advanced age [26] and postoperative infection were independent predictors of NOPAF following pulmonary resection [17]. Advanced age may lead to remodeling of cardiac structures that initiate and maintain the atrial fibrillation re-entry circuit [27]. Our findings also suggested that advanced age is an independent risk factor for NOPAF according to multivariable logistic regression analysis. Unlike previous studies, however, some risk parameters, such as male, ischemic heart disease, extent of resection, and surgical approach, were not significantly associated with NOPAF [25].

The relationship between lung function (%MVV) and NOPAF has not been clearly defined in pulmonary resection patients. Lung function (%MVV), in our study, is one of the prominent protective factors for the development of NOPAF, and epidemiological studies have demonstrated a progressive increase in the prevalence of AF with reduced FEV1 and MVV. The relationship between pulmonary function and atrial fibrillation has been studied in the atherosclerosis risk community cohort [28]. In the Atherosclerosis Risk in Communities (ARIC) study, reduced FEV1 and obstructive airway disease were associated with a higher incidence of AF. A prospective study including 13,430 patients demonstrated that reduced FEV1 was an independent predictor of new onset atrial fibrillation [29]. These findings are very similar to our conclusions and support the association between poor lung function and high incidence of AF. Although %FEV1 (OR, 0.98; 95% CI, 0.97-1.00; *p*=0.086) did not enter the final prediction model in our study, its difference between the two groups was still statistically significant (*P*< 0.05).

The specific mechanism of decreased lung function and increased incidence of AF is still unclear. The ectopic beats that cause AF are more likely to originate from the pulmonary vein wall, where they are integrated with the atrium. This ectopic beat may be caused by changes in gas composition or pulmonary hypertension [30, 31], which leads to elevated atrial pressure, altering the electrophysiological properties of atrial tissues and then initiating atrial fibrillation [32]. Emphysema generally affects right ventricular function, but some studies have suggested that emphysema can also affect left ventricular function, which is mainly associated with left ventricular hypertrophy [33], diastolic dysfunction [34], narrowing of pulmonary vein diameter, and structural/electrical abnormalities in pulmonary vein region [35]. Pulmonary vein fibrosis [36] and pulmonary vein stretch caused by increased atrial pressure might also have an impact on new-onset atrial fibrillation in patients with reduced pulmonary function [37]. The above mechanisms are still theoretical speculations and need to be explored and verified by further molecular biological experiments.

Of note, this study found that laterality (right and left) was a prominent risk factor for the development of NOPAF. Bilateral lung surgery (laterality: right and left) (OR, 30.07; 95% CI, 8.02-112.75; *P*<0.001) significantly increased the incidence rate of NOPAF. We speculated that laterality (right and left) may lead to increased pain-related sympathetic stimulation, extensive local tissue trauma and an enhanced inflammatory response from bilateral skin incisions and intercostal muscle injury, which ultimately leads to a significant increase in the incidence of NOPAF. The specific pathogenesis requires further research and exploration. In addition, we found an interesting phenomenon that admission heart rate (OR, 0.97; 95% CI, 0.94-1.00; *P* < 0.05) is a protective factor of NOPAF, which differs from previous literature reports and deserves further research.

To the best of our knowledge, this study was the first nomogram model to predict NOPAF in patients underwent pulmonary resection. However, our results also have obvious limitations. First, our model based on retrospective data is susceptible to biases. Second, since this was a single institution clinical study with only 54 NOPAF cases, hence, the outcomes of the research are not representative of the general population. Third, given that we could not determine the duration of NOPAF episodes, therefore, underestimation is possible. In addition, cases of subclinical or paroxysmal AF may be misreported at clinical visits due to the lack of typical clinical symptoms. Consequently, it is acceptable to underestimate the true incidence of NOPAF. Last, the failure to detect statistical significance of FEV1, number of lymph nodes dissected and LVEF may be due to the relatively small number of cases in each subgroup.

## Conclusion

In summary, A nomogram, composed of five independent predictors, namely age, admission heart rate, extent of resection, laterality, %MVV, was constructed, which may assist clinicians predict the individual probability of NOPAF and perform available prophylaxis.

## Data Availability

The data that support the findings of this study are available from the corresponding author upon reasonable request.

## Abbreviations

NOPAF: New-onset postoperative atrial fibrillation
%MVV: Percent maximum ventilation volume per minute
C-index: Concordance index
AF: Atrial fibrillation
BMI: Body mass index
USTC: University of Science and Technology of China
%FEV1: percent forced expiratory volume in 1 second
LVEF: Left ventricular ejection fraction
LAD: Left atrial anteroposterior diameter
LVEDV: Left ventricular end-diastolic volume
LVESV: Left ventricular end-systolic volume
NYHA: New York Heart Association
VATS: Video-assisted thoracic surgery
ROC: area under the receiver operating characteristic
DCA: Decision curve analysis

## References

[1] Fan K, Chen L, Liu F, Ding X, Yan P, Gao M, Yu W, Liu H, Yu Y (2022). Predicting New-Onset Postoperative Atrial Fibrillation Following Isolated Coronary Artery Bypass Grafting: Development and Validation of a Novel Nomogram. Int J Gen Med.

[2] Bidar E, Bramer S, Maesen B, Maessen JG, Schotten U (2013). Post-operative Atrial Fibrillation - Pathophysiology, Treatment and Prevention. J Atr Fibrillation.

[3] Roselli EE, Murthy SC, Rice TW, Houghtaling PL, Pierce CD, Karchmer DP, Blackstone EH (2005). Atrial fibrillation complicating lung cancer resection. J Thorac Cardiovasc Surg.

[4] Amar D, Zhang H, Chung MK, Tan KS, Desiderio D, Park BJ, Pedoto A, Roistacher N, Isbell JM, Molena D (2022). Amiodarone with or without N-Acetylcysteine for the Prevention of Atrial Fibrillation after Thoracic Surgery: A Double-blind. Randomized Trial. Anesthesiol.

[5] Siontis KC, Gersh BJ, Weston SA, Jiang R, Kashou AH, Roger VL, Noseworthy PA, Chamberlain AM (2020). Association of new-onset atrial fibrillation after Noncardiac surgery with subsequent stroke and transient ischemic attack. Jama.

[6] Jiang J, He M, Xu Y (2021). preoperative electrocardiogram and perioperative methods for predicting new-onset atrial fibrillation during lung surgery. J Cardiothorac Vasc Anesth.

[7] Kashiwagi M, Hirai Y, Kuroi A, Ohashi T, Yata Y, Fusamoto A, Iguchi H, Higashimoto N, Tanimoto T, Tanaka A, et al. Relationship between postoperative atrial fibrillation and its recurrence after lung resection. Surg Today. 2023;53(10):1139-48. 10.1007/s00595-023-02670-4 36894737

[8] Nattel S (2002). New ideas about atrial fibrillation 50 years on. Nature.

[9] Lomivorotov VV, Efremov SM, Pokushalov EA, Karaskov AM (2016). New-onset atrial fibrillation after cardiac surgery: pathophysiology, prophylaxis, and treatment. J Cardiothorac Vasc Anesth.

[10] Rezaei Y, Peighambari MM, Naghshbandi S, Samiei N, Ghavidel AA, Dehghani MR, Haghjoo M, Hosseini S (2020). Postoperative atrial fibrillation following cardiac surgery: from pathogenesis to potential therapies. Am J Cardiovasc Drugs.

[11] Dobrev D, Aguilar M, Heijman J, Guichard JB, Nattel S (2019). Postoperative atrial fibrillation: mechanisms, manifestations and management. Nat Rev Cardiol.

[12] Wu DH, Xu MY, Mao T, Cao H, Wu DJ, Shen YF (2012). Risk factors for intraoperative atrial fibrillation: a retrospective analysis of 10,563 lung operations in a single center. Ann Thorac Surg.

[13] Zhou Q, Hu J, Guo Y, Zhang F, Yang X, Zhang L, Xu X, Wang L, Wang H, Hou Y (2013). Effect of the stellate ganglion on atrial fibrillation and atrial electrophysiological properties and its left-right asymmetry in a canine model. Exp Clin Cardiol.

[14] Acikel S, Bozbas H, Gultekin B, Aydinalp A, Saritas B, Bal U, Yildirir A, Muderrisoglu H, Sezgin A, Ozin B (2008). Comparison of the efficacy of metoprolol and carvedilol for preventing atrial fibrillation after coronary bypass surgery. Int J Cardiol.

[15] Iwata T, Nagato K, Nakajima T, Suzuki H, Yoshida S, Yoshino I (2016). Risk factors predictive of atrial fibrillation after lung cancer surgery. Surg Today.

[16] Bagshaw SM, Galbraith PD, Mitchell LB, Sauve R, Exner DV, Ghali WA (2006). Prophylactic amiodarone for prevention of atrial fibrillation after cardiac surgery: a meta-analysis. Ann Thorac Surg.

[17] Garner M, Routledge T, King JE, Pilling JE, Veres L, Harrison-Phipps K, Bille A, Harling L (2017). New-onset atrial fibrillation after anatomic lung resection: predictive factors, treatment and follow-up in a UK thoracic centre. Interact Cardiovasc Thorac Surg.

[18] Tang Y, Chen Q, Zha L, Feng Y, Zeng X, Liu Z, Li F, Yu Z (2021). Development and validation of nomogram to predict long-term prognosis of critically ill patients with acute myocardial infarction. Int J Gen Med.

[19] Coutant C, Olivier C, Lambaudie E, Fondrinier E, Marchal F, Guillemin F, Seince N, Thomas V, Levêque J, Barranger E (2009). Comparison of models to predict nonsentinel lymph node status in breast cancer patients with metastatic sentinel lymph nodes: a prospective multicenter study. J Clin Oncol.

[20] Fitzgerald M, Saville BR, Lewis RJ (2015). Decision curve analysis. Jama.

[21] Ishibashi H, Wakejima R, Asakawa A, Baba S, Nakashima Y, Seto K, Kobayashi M, Okubo K (2020). Postoperative atrial fibrillation in lung cancer lobectomy-analysis of risk factors and prognosis. World J Surg.

[22] Gómez-Caro A, Moradiellos FJ, Ausín P, Díaz-Hellín V, Larrú E, Pérez-Antón JA (2006). Martín de Nicolás JL: Risk factors for atrial fibrillation after thoracic surgery. Arch Bronconeumol.

[23] Dyszkiewicz W, Skrzypczak M (1998). Atrial fibrillation after surgery of the lung: clinical analysis of risk factors. Eur J Cardiothorac Surg.

[24] Brundel B, Ai X, Hills MT, Kuipers MF, Lip GYH, de Groot NMS (2022). Atrial fibrillation. Nat Rev Dis Primers.

[25] Mansour Z, Kochetkova EA, Santelmo N, Meyer P, Wihlm JM, Quoix E, Massard G (2009). Risk factors for early mortality and morbidity after pneumonectomy: a reappraisal. Ann Thorac Surg.

[26] Díez-Villanueva P, Alfonso F (2019). Atrial fibrillation in the elderly. J Geriatr Cardiol.

[27] Cheniti G, Vlachos K, Pambrun T, Hooks D, Frontera A, Takigawa M, Bourier F, Kitamura T, Lam A, Martin C (2018). Atrial fibrillation mechanisms and implications for catheter ablation. Front Physiol.

[28] Li J, Agarwal SK, Alonso A, Blecker S, Chamberlain AM, London SJ, Loehr LR, McNeill AM, Poole C, Soliman EZ (2014). Airflow obstruction, lung function, and incidence of atrial fibrillation: the Atherosclerosis Risk in Communities (ARIC) study. Circulation.

[29] Buch P, Friberg J, Scharling H, Lange P, Prescott E (2003). Reduced lung function and risk of atrial fibrillation in the Copenhagen City Heart Study. Eur Respir J.

[30] Olsson SB (2001). Atrial fibrillation–Where do we stand today?. J Intern Med.

[31] Haïssaguerre M, Jaïs P, Shah DC, Takahashi A, Hocini M, Quiniou G, Garrigue S, Le Mouroux A, Le Métayer P, Clémenty J (1998). Spontaneous initiation of atrial fibrillation by ectopic beats originating in the pulmonary veins. N Engl J Med.

[32] Alonso A, Tang W, Agarwal SK, Soliman EZ, Chamberlain AM, Folsom AR (2012). Hemostatic markers are associated with the risk and prognosis of atrial fibrillation: the ARIC study. Int J Cardiol.

[33] Smith BM, Kawut SM, Bluemke DA, Basner RC, Gomes AS, Hoffman E, Kalhan R, Lima JA, Liu CY, Michos ED (2013). Pulmonary hyperinflation and left ventricular mass: the Multi-Ethnic Study of Atherosclerosis COPD Study. Circ.

[34] Govindarajulu US, Spiegelman D, Thurston SW, Ganguli B, Eisen EA (2007). Comparing smoothing techniques in Cox models for exposure-response relationships. Stat Med.

[35] Smith BM, Prince MR, Hoffman EA, Bluemke DA, Liu CY, Rabinowitz D, Hueper K, Parikh MA, Gomes AS, Michos ED (2013). Impaired left ventricular filling in COPD and emphysema: is it the heart or the lungs? The multi-ethnic study of Atherosclerosis COPD Study. Chest.

[36] Everett THt, Olgin JE (2007). Atrial fibrosis and the mechanisms of atrial fibrillation. Heart Rhythm.

[37] Kalifa J, Jalife J, Zaitsev AV, Bagwe S, Warren M, Moreno J, Berenfeld O, Nattel S (2003). Intra-atrial pressure increases rate and organization of waves emanating from the superior pulmonary veins during atrial fibrillation. Circ.

